# Adrenal Lymphangioma Masquerading as a Catecholamine Producing Tumor

**DOI:** 10.1155/2015/380151

**Published:** 2015-11-05

**Authors:** Israel Hodish, Lindsay Schmidt, Andreas G. Moraitis

**Affiliations:** ^1^Division of Internal Medicine, University of Michigan Medical Center, Ann Arbor, MI 48109, USA; ^2^Department of Pathology, University of Michigan, Ann Arbor, MI 48109, USA; ^3^Corcept Therapeutics, Menlo Park, CA 94025, USA

## Abstract

*Objective*. To report the unusual case of an adrenal lymphangioma presenting in a patient with an adrenal cystic lesion and biochemical testing concerning for pheochromocytoma. The pertinent diagnostic and imaging features of adrenal lymphangiomas are reviewed. *Methods*. We describe a 59-year-old patient who presented with hyperhidrosis and a 2.2 by 2.2 cm left adrenal nodule. Biochemical evaluation revealed elevated plasma-free normetanephrine, urine normetanephrine, urine vanillylmandelic acid, and urine norepinephrine levels. Elevated plasma norepinephrine levels were not suppressed appropriately with clonidine administration. *Results*. Given persistent concern for pheochromocytoma, the patient underwent adrenalectomy. The final pathology was consistent with adrenal lymphangioma. *Conclusions*. Lymphangiomas are benign vascular lesions that can very rarely occur in the adrenal gland. Imaging findings are generally consistent with a cyst but are nonspecific. Excluding malignancy in patients presenting with adrenal cysts can be difficult. Despite its benign nature, the diagnosis of adrenal lymphangioma may ultimately require pathology.

## 1. Introduction

Lymphangiomas are benign vascular lesions that most commonly occur in the head, neck, and axilla [1]. Lymphangiomas of the adrenal gland are very rare, with an estimated incidence of 0.064 to 0.18 percent in autopsy series [2]. These lesions are generally asymptomatic but may be found incidentally on radiographic imaging during work-up for unrelated conditions. Therefore, it is important to distinguish these benign cysts from malignant adrenal lesions. Here, we present the diagnostic dilemma of a patient who presented with an adrenal cystic lesion and biochemical evaluation concerning for pheochromocytoma. The patient was ultimately found to have an adrenal lymphangioma.

## 2. Case Report

A 59-year-old male with past medical history of hypertension, obesity, hyperhidrosis, and secondary polycythemia presented to his outpatient hematologist in 2009 for evaluation of hyperhidrosis. The patient endorsed several-year history of profuse episodic sweating that interfered with his social interactions. He denied any headaches or palpitations. The patient had a known history of 1.7 by 1.4 cm adrenal nodule discovered incidentally on MR scan performed that same year. The nodule did not enhance, and imaging characteristics were most consistent with a cyst.

Biochemical evaluation in 2009 included an elevated 24-hour urine normetanephrine, norepinephrine, and vanillylmandelic acid as indicated in Table 1. Urine 24-hour metanephrine and epinephrine were both within normal limits. The patient was not taking any medications that could interfere with the measurement of catecholamines or metanephrines. In 2010, the patient underwent an I^123^metaiodobenzylguanidine (MIBG) scan that was negative for pheochromocytoma.

Given continued symptoms of hyperhidrosis, the patient was referred to endocrinology in 2013. Repeat biochemical evaluation at that time was notable for an elevated plasma-free normetanephrine, plasma norepinephrine, 24-hour urine normetanephrine, and 24-hour urine norepinephrine as documented in Table 1. Plasma-free metanephrine, plasma epinephrine, urine 24-hour metanephrine, and urine 24-hour epinephrine were within normal limits. He was also diagnosed with new onset diabetes.

**Figure 1:**
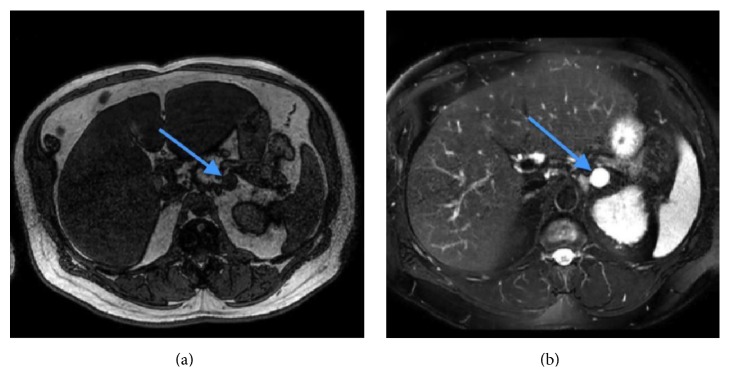
MRI of the adrenals. Within the lateral limb of the left adrenal gland, there is a 2.2 × 2.2 cm lesion (blue arrows) with uniformly high signal intensity on T2-weighted sequences (b) and low signal on T1-weighted images (a) with no significant loss of signal on opposed phase relative to in phase images.

With regard to further imaging studies, adrenal protocol CT demonstrated a 2.1 by 2.2 cm left adrenal nodule which could not be classified as lipid rich adenoma. The density measurement of the nodule was 12 Hounsfield units before contrast and 22 Hounsfield units on the enhanced study. The absolute enhancement washout value was 20%, below the 60% threshold for an adenoma. Adrenal protocol MR demonstrated a 2.2 × 2.2 cm lesion with uniformly high signal intensity on T2-weighted sequences and low signal on T1-weighted images with no enhancement on contrast administration. MR findings were most consistent with a cyst (Figure 1).

Given the patient's modestly elevated normetanephrine and norepinephrine, confirmatory testing was performed with a clonidine suppression test. Norepinephrine was 891 pg/mL (normal reference range < 500 pg/mL) prior to 0.3 mg clonidine administration and 708 pg/mL 3 hours after clonidine administration. Normetanephrine was 0.95 nmol/L (normal reference range < 0.90 nmol/L) prior to clonidine administration and 0.94 nmol/L 3 hours after clonidine administration which is suggestive of pheochromocytoma.

**Figure 2:**
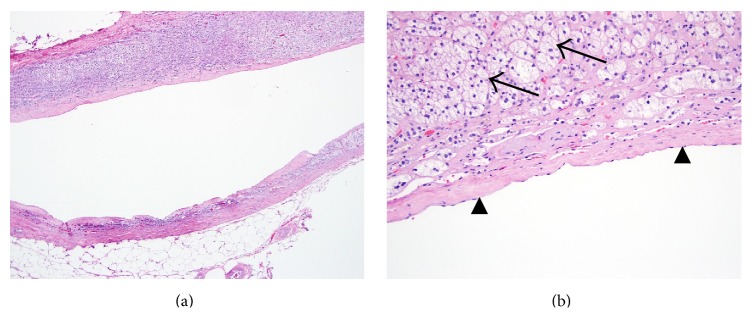
(a) Gross pathology demonstrated a 2 cm fluid-filled cyst. Hematoxylin and eosin stain at low magnification demonstrates a smooth-lined cyst. (b) Hematoxylin and eosin stain at high magnification shows endothelial cells lining the wall of the cystic lesion (black arrowheads). The stroma contains foamy cells (black arrows) with some mononuclear infiltrates. The entire picture is most consistent with benign vascular cyst.

Due to persistent concern for potential pheochromocytoma with cystic degeneration, he was started on phenoxybenzamine and underwent laparoscopic left adrenalectomy without complications. Surgical pathology demonstrated a benign vascular cyst consistent with lymphangioma (Figure 2).

Repeat plasma fractionated metanephrines performed after surgery were within normal limits. Incidentally, patient reported significant improvement in his hyperhidrosis after surgery and also significant weight loss and improvement of diabetes control. Polycythemia was most likely not related to the adrenal tumor, since no changes have been noticed up to 6-month postop follow-up.

## 3. Discussion

Adrenal cysts can be classified as pseudocysts, endothelial cysts, epithelial cysts, or parasitic cysts. The estimated frequency of each subtype varies by series, with approximately 39–78% classified as pseudocysts, 20–45% endothelial cysts, 2–9% epithelial cysts, and 0–7% parasitic cysts [3–5]. Endothelial cysts can be further divided into lymphangiomatous and angiomatous cysts. Lymphangiomas are believed to arise from faulty lymphatic development leading to either isolation of the lymphangioma from larger lymphatic channels or lack of fusion with the venous system [1, 4, 6].

An estimated 7–15% of adrenal cysts are associated with malignancy [3, 5, 7, 8]. Although lymphangiomas are benign lesions, aspiration or surgical removal becomes necessary when malignancy cannot be excluded based on imaging alone. On ultrasonography, lymphangiomas generally present as well-demarcated multiloculated cysts. Less commonly, they present as unicameral cysts [9, 10]. On CT scan, they display capsular enhancement with internal attenuation values generally in the range of water [9–11]. On MR, these cysts are hypointense and nonenhancing on T1-weighted images and hyperintense on T2-weighted images [11]. However, the sensitivity and specificity of these imaging modalities for excluding malignancy remain unknown. In our case the mild postcontrast enhancement reported on the adrenal CT scan was primarily enhancement of the rim of the cystic lesion specifically from the part of the lesion abutting the normal adrenal tissue. Including the entire surface of the lesion on HU density measurement is advisable when there is suspicion of pheochromocytoma with cystic degeneration. Postcontrast HU of the lesion excluding the rim of the lesion showed no enhancement.

Identification of surgically excised adrenal cysts can be confirmed histologically. Adrenal lymphangiomas are characterized by multicystic architecture with a simple endothelial lining. On immunohistochemistry, these lesions stain negative for keratin and positive for D2-40, a marker of lymphatic endothelium [4].

Diagnosis in this case was complicated by biochemical testing that was concerning for possible functional adrenal tumor. Given the low prevalence of pheochromocytoma in the general population, even screening tests with high specificity will have more false-positive results than true-positive results [12, 13]. In cases where there is high clinical suspicion for pheochromocytoma, plasma-free metanephrines and normetanephrines are an appropriate first screening test given their high reported sensitivity of 96–99% [13, 14]. Large elevations in plasma metanephrines or normetanephrines several times the upper limit of normal are highly suggestive of pheochromocytoma and should prompt further investigation to localize the tumor [12–15]. In cases where plasma metanephrine or normetanephrine elevations are mild, further testing can be performed to help confirm or exclude the diagnosis including plasma catecholamines, urinary fractionated metanephrines, urinary catecholamines, and vanillylmandelic acid. Factors that can interfere with the diagnostic accuracy of these tests should also be addressed. Physiologic stress, obstructive sleep apnea, caffeine, nicotine, and several medications including many antihypertensives, antidepressants, stimulants, and sympathomimetics can all interfere with test results and lead to false-positive results [13].

In cases where biochemical test results are equivocal, a clonidine suppression test can be used to help clarify the diagnosis. Clonidine is a central alpha2-agonist that suppresses catecholamine release by the sympathetic nervous system. Release of catecholamines from a pheochromocytoma is thought to be autonomous and therefore would not be suppressed by clonidine [16]. Although several criteria have been proposed to define an appropriate clonidine response, a 3-hour postclonidine plasma norepinephrine level of less than 500 pg/mL with a decline of at least 50% has been used with a sensitivity and specificity of 97% and 74%, respectively [17]. The specificity of the test is significantly reduced in patients with a preclonidine plasma norepinephrine level in the normal range [18]. Yet, even using the much more specific criteria which define an abnormal test result simply as a plasma norepinephrine level of 500 pg/mL or less after clonidine administration (specificity of 96%) [17], our patient still had an abnormal clonidine response. Beta-blockers, tricyclic antidepressants, and thiazide diuretics have all been reported to produce false-positive results; however, none of these medications are applicable to our patient. This case illustrates the difficulty in ruling out pheochromocytoma in patients with benign adrenal lesions.

Adrenal cysts are usually asymptomatic, although local symptoms can vary with the size and position of the lesion. In one case series of 9 patients with adrenal lymphangioma 44% (4/9) presented with abdominal, flank, or back pain while an additional 44% (4/9) were asymptomatic and found incidentally [4]. In the same series one case was found on work-up for labile hypertension that reportedly normalized after resection [19]. Interestingly, our patient reported resolution of his hyperhidrosis after surgery. However, given that adrenal lymphangiomas are nonfunctional cysts, the mechanism by which this could be tied to his hyperhidrosis remains unclear.

## 4. Conclusion

Here, we present the diagnostic dilemma of a patient with a cystic adrenal lesion in the setting of laboratory testing concerning for pheochromocytoma. Work-up included an elevated plasma norepinephrine level that was not not suppressed appropriately with clonidine administration. The patient ultimately underwent adrenalectomy that revealed an adrenal lymphangioma. This case illustrates the difficulty of definitely excluding pheochromocytoma in a patient with a rare benign adrenal cyst.

## Figures and Tables

**Table 1:** Patient's catecholamine levels before and after left adrenalectomy.

	2009 (Preoperative)	Reference range	2013 (Preoperative)	2013 (Postoperative)	Reference range
Plasma-free normetanephrine	nd	nd	**1.6 nmol/L**	0.53 nmol/L	<0.90 nmol/L
Plasma-free metanephrine	nd	nd	<0.20 nmol/L	<0.20 nmol/L	<0.50 nmol/L
Plasma norepinephrine	nd	nd	**891 pg/mL**	**659 pg/mL**	0–500 pg/mL
Plasma epinephrine	nd	nd	52 pg/mL	24 pg/mL	0–100 pg/mL
Urine normetanephrine	1868 ***μ*** **g**/24 **h** **o** **u** **r** **s**	110–1050 *μ*g/24 hours	1123 ***μ*** **g**/24 **h** **o** **u** **r** **s**	nd	50–800 *μ*g/24 hours
Urine metanephrines	254 *μ*g/24 hours	35–460 *μ*g/24 hours	123 *μ*g/24 hours	nd	0–300 *μ*g/24 hours
Urine norepinephrine	414 ***μ*** **g**/24 **h** **o** **u** **r** **s**	0–140 *μ*g/24 hours	257 ***μ*** **g**/24 **h** **o** **u** **r** **s**	nd	0–100 *μ*g/24 hours
Urine epinephrine	16 *μ*g/24 hours	0–32 *μ*g/24 hours	10.2 *μ*g/24 hours	nd	0–20 *μ*g/24 hours
Urine vanillylmandelic acid	9.6 ***μ*** **g**/24 **h** **o** **u** **r** **s**	0.0–7.5 *μ*g/24 hours	nd	nd	nd

nd: not done.

Results outside the reference range are indicated in bold.
